# Pharmacologically Induced Ex Copula Ejaculation in Horses and Donkeys

**DOI:** 10.3389/fvets.2021.669423

**Published:** 2021-12-01

**Authors:** Afroza Khanam, Ayman A. Swelum, Firdous A. Khan

**Affiliations:** ^1^Department of Large Animal Medicine and Surgery, School of Veterinary Medicine, St. George's University, True Blue, Grenada; ^2^Department of Theriogenology, Faculty of Veterinary Medicine, Zagazig University, Zagazig, Egypt; ^3^Department of Animal Production, College of Food and Agriculture Sciences, King Saud University, Riyadh, Saudi Arabia

**Keywords:** equine, imipramine, xylazine, detomidine, semen

## Abstract

Pharmacologically induced ex copula ejaculation is a method used for collection of semen when the traditional methods of semen collection are not feasible. Common indications for this method include health issues that either preclude the physical act of mating or result in impaired erection and ejaculation. The method also offers an alternative when there is a lack of equipment and facilities required for semen collection using the conventional artificial vagina method. A variety of pharmacological protocols for ex copula ejaculation in stallions have been evaluated in both research and clinical settings with no serious side-effects reported. In general, these protocols included tricyclic antidepressants, alpha-2 adrenergic receptor agonists, and smooth muscle stimulators, either individually or in combination. Although there is a lot of variation in the ejaculatory rates among studies, a combination of imipramine and xylazine appears to be an effective option for inducing ejaculation in stallions. In cases where this protocol is not successful, collections should be reattempted using a combination of imipramine, detomidine, and oxytocin. Regardless of the protocol used, a quiet environment with minimal disturbance is associated with a better outcome. In contrast to the body of literature available on pharmacologically induced ex copula ejaculation in horses, only a few studies have been published so far on this topic in donkeys. Further studies are warranted to investigate whether pharmacologically induced ex copula ejaculation is an effective method of semen collection in jacks and to compare different pharmacological protocols for inducing ejaculation in jacks.

## Introduction

Semen collection in horses is commonly performed using an artificial vagina with the stallion mounting either a jump mare or a phantom/dummy. Pharmacologically induced ex copula ejaculation is an alternative method of semen collection used in stallions with musculoskeletal, neurological, or behavioral abnormalities that preclude the physical act of mounting and copulation or interfere with erection and ejaculation. Documented clinical examples include stallions with lameness (1), disability due to multiple causes including chronic obstructive pulmonary disease, cardiac disease, and chronic musculoskeletal disease of the forelimbs (2), paraphimosis and erectile dysfunction (3), idiopathic ejaculatory dysfunction (4) and ejaculatory failure associated with aortic-iliac thrombosis (5). Several research studies have been conducted in the past to evaluate different pharmacological protocols for inducing ejaculation in stallions and this topic continues to be an area of active research. In contrast to horses, pharmacologically induced ex copula ejaculation has not been investigated extensively in donkeys, and results from the few studies reported so far are inconsistent. The present article aims to review the published literature on pharmacologically induced ex copula ejaculation in horses and donkeys.

## Pharmacologically Induced Ex Copula Ejaculation in Horses

A variety of drugs and their combinations have been used to induce ex copula ejaculation in horses. Broadly speaking, these drugs include tricyclic antidepressants, alpha-2 adrenergic receptor agonists, and smooth muscle stimulators. As discussed in the subsections below, several protocols have been evaluated with respect to their efficacy in inducing ejaculation in stallions.

### Imipramine Alone Protocol

The initial attempts at ex copula ejaculation in stallions were based on the previously documented effects of antidepressants such as imipramine and clomipramine on sexual excitement and ejaculation in men (6, 7). These effects are attributed to imipramine- or clomipramine-mediated inhibition of norepinephrine reuptake, resulting in promotion of alpha-adrenergic activity (8). In a study involving five male horses (one inexperienced young stallion, two mature normal breeding stallions, one 5-year-old stallion with erection and ejaculatory dysfunction, and one long-term gelding), imipramine hydrochloride administered orally at a dose range of 100–600 mg twice a day resulted in repeated erection and masturbation (9). In the same study, the authors reported a similar response when imipramine was administered intravenously over a dose range of 50–1,000 mg. Moreover, the intravenous treatment resulted in ejaculation in 3 out of 28 treatment attempts (11% ejaculation rate) in the four intact stallions. The ejaculations were observed in one of the two mature stallions and the stallion with erection and ejaculatory dysfunction. No serious side effects were reported in either oral or intravenous imipramine treatments. However, higher doses were associated with mild ataxia and drowsiness. Apart from the study by McDonnell et al. (9), the use of imipramine alone for inducing ejaculation in stallions has not received any attention, presumably owing to the low ejaculation rate observed with this protocol.

### Xylazine Alone Protocol

Xylazine is an α2-adrenergic agonist that mediates its effects mainly through stimulation of central α2-adrenergic receptors. This stimulation decreases the neurotransmission of norepinephrine and dopamine (10). The administration of xylazine alone for ex copula ejaculation in horses has been reported in both research (11, 12) and clinical settings (5). In a study involving 28 stallions (12 horses and 16 ponies), xylazine was administered intravenously at a dose rate of 0.7 mg/kg and the stallions were left undisturbed for 30 min (11). Each stallion underwent a trial with sexual prestimulation and a trial without sexual prestimulation. Ejaculation was reported in 15 (27%) of the 56 trials. Eleven of the ejaculations were reported with sexual prestimulation and four without sexual prestimulation, suggesting a beneficial effect of sexual prestimulation in this protocol. A much higher ejaculation rate [10 out of 11 attempts (91%)] after xylazine administration was reported in a case report involving two stallions with ejaculatory failure associated with aortic-iliac thrombosis (5). The authors attributed the higher ejaculation rate to relatively longer periods of prestimulation used in the clinical cases and to a speculated hypersensitivity to alpha adrenergic stimulation in stallions with ejaculatory dysfunction. In a more recent study involving 12 mature stallions (12), xylazine was administered intravenously at a dose rate of 0.7 mg/kg and each stallion underwent two trials. Ejaculation was reported in 4 of 24 trials (17%). Absence of sexual prestimulation might have contributed to the lower ejaculation rate in this study. Semen quality of the ejaculates induced by xylazine was reported to be mostly similar to the ejaculates collected from the stallions using artificial vagina (11, 12). Apart from mild to heavy standing sedation (11, 12), no other side effects were reported in any of the studies with this protocol.

### Imipramine and Xylazine Combination Protocol

As discussed above, both imipramine and xylazine promote alpha-adrenergic activity. Several studies have investigated the effectiveness of combined treatment with imipramine and xylazine in causing ex copula ejaculation in stallions. In a study involving 8 sexually experienced pony stallions (13), imipramine hydrochloride was administered intravenously at a dose rate of 2 mg/kg followed 10 min later by intravenous injection of 0.3 mg/kg xylazine hydrochloride. In a total of 48 trials, each stallion underwent six treatment trials conducted at 4-day intervals and three of the six trials involved sexual prestimulation using an ovariectomized pony mare. The combined treatment resulted in erection, masturbation, and ejaculation in 34 (71%), 25 (52%), and 16 (33%) of the 48 trials. Out of the 16 ejaculations, six occurred within the 10 min after imipramine administration or before xylazine administration. Sexual prestimulation did not appear to have a beneficial effect. On the contrary, there was a tendency for a reduced likelihood of ejaculation in the trials that were preceded by sexual stimulation (25% with prestimulation vs. 42% without prestimulation).

In another study (14) using a combination of imipramine and xylazine in five sexually experienced pony stallions, ejaculation was observed in 9 (53%) out of 17 trials. A difference between this study and the previous one (13) was that xylazine was administered only if ejaculation did not occur within 60 min of imipramine administration. Seven ejaculations occurred within that timeframe and two ejaculations occurred after the administration of xylazine. Another difference was that the interval between trials in this study was shorter (2–3 days) than the interval (4 days) in the previous study. The authors suggested that the higher ejaculation rate may be attributed in part to lesser disturbance after treatment in the latter study.

Although imipramine is the most frequently used antidepressant for ex copula ejaculation in horses, clomipramine administered intravenously at a dose rate of 2.2 mg/kg followed 55 min later by xylazine (0.5 mg/kg intravenously) has also been reported to induce ejaculation in a stallion with fractured radius (1).

Oral administration of imipramine combined with intravenous administration of xylazine has also been tested in multiple studies. Johnston and DeLuca (15) in a study involving six stallions administered imipramine at a dose rate of 0.8–2.5 mg/kg followed 1–3 h later by intravenous injection of xylazine (0.3 mg/kg intravenously). Ejaculation was reported in six out of 14 trials (57%). In more recent studies, imipramine hydrochloride was administered orally at a dose rate of 3 mg/kg followed 2 h later by intravenous administration of xylazine hydrochloride at a dose rate of 0.7 mg/kg (12, 16). The former study (16) involved eight sexually mature stallions (six ponies and two horses). Each stallion underwent eight trials at intervals of 2–3 days. Ejaculation was reported in 44 of the 64 trials (68%). The latter study (12) involved 12 mature stallions and an additional half dose of xylazine was administered if ejaculation did not occur within 10 min of the first xylazine injection. Ejaculation was reported in seven of 24 trials (29%). An ejaculation rate of 33% (five out of 15 attempts) was reported after oral administration of imipramine hydrochloride (3 mg/kg) followed 2 h later by intravenous injection of xylazine (0.25 mg/kg) in a 20-year-old Quarter Horse with paraphimosis secondary to priapism (3).

In general, ejaculates collected using the imipramine and xylazine combination had higher sperm concentration, higher total sperm number, lower total volume and pH, and similar total and progressive motility when compared with the baseline in copula ejaculates from the stallions (13, 14, 16). Side effects reported with the protocol included sialorrhea (12), mild hemolysis (13), and mild to heavy standing sedation (12, 13).

### Detomidine-Based Protocols

Similar to xylazine, detomidine is an alpha-adrenergic agonist that has been tested with respect to its effectiveness in inducing ejaculation in horses. In a study involving 12 mature stallions (12), detomidine administered alone (0.02 mg/kg intravenously) or 2 h after administration of imipramine (3 mg/kg orally) did not result in any ejaculations from 24 trails of each treatment (0%). However, when administered in combination with oxytocin (20 IU intravenously) or imipramine (3 mg/kg orally) and oxytocin (20 IU intravenously), ejaculation was reported in two out of 24 attempts (8.3%) and seven out of 24 trials (29%), respectively. The combination of imipramine, detomidine, and oxytocin was also reported to be effective in a 7-year-old Quarter Horse stallion with ejaculatory dysfunction (4). Detomidine alone was reported to be effective in causing ex copula ejaculations in a 4-year-old pony stallion (17). After sexual prestimulation, detomidine hydrochloride was administered (0.02 mg/kg intravenously) followed 5 min later by a second injection (0.01 mg/kg). The authors reported seven ejaculations in the stallion using this approach. This contrasts with the lack of ejaculation with detomidine alone reported by Cavalero et al. (12). The difference may be attributed to the sexual prestimulation used before detomidine administration by Rowley et al. (17).

Ejaculates collected using a combination of detomidine and oxytocin or a combination of imipramine, detomidine and oxytocin had lower volume and higher sperm concentration than in copula ejaculates. Apart from a mild standing sedation, no other side effects were reported during or after detomidine-induced ex copula ejaculation (12).

### Prostaglandin F2alpha-Based Protocol

Prostaglandin F2alpha (PGF2α), a smooth muscle stimulator, has been tested in a study that involved eight pony stallions (18). Based on individual stallion titration trials, a dose range of 0.01–0.15 mg/kg intramuscularly resulted in ejaculation in 75% of the collection attempts. However, this protocol is not used commonly due to the side effects associated with PGF2α treatment (abdominal cramping, sweating, and urine dripping), resulting in discomfort to the stallion and increased chances of urine contamination of the semen (16).

## Pharmacologically Induced Ex Copula Ejaculation in Donkeys

Semen collection in jacks (male donkeys) is performed mainly for artificial insemination of jennies or mares either immediately after the collection or after cooling or cryopreservation of the collected semen. Apart from being an important part of the breeding soundness evaluation, semen collection aids in diagnosis and prevention of venereal diseases (19). Jacks can be collected using an artificial vagina, similar to stallions (20). However, compared to stallions, jacks are more difficult to train to mount a phantom because of a high latency to erection and mounting (21). Therefore, pharmacologically induced ex copula ejaculation could be a useful alternative to traditional methods of semen collection in jacks. To date, only four studies have investigated ex copula ejaculation in jacks (19, 22–24). The pharmacological regimens used in these studies to induce ejaculation included imipramine, butorphanol, xylazine, and detomidine.

Using oral administration of imipramine hydrochloride (3 mg/kg) followed 2 h later by intravenous injection of xylazine hydrochloride (1.1 mg/kg) in five jacks, Naoman and Ali (23) reported 29 ejaculates from 30 trials (96.6%). The collected ejaculates were of good quality and the interval from xylazine injection to ejaculation ranged from 5 to 10 min to 1 h, presumably due to biological variation and different arousal levels of the animals. In contrast to the high success rate reported by Naoman and Ali (23), extremely poor responses were reported in three other studies (19, 22, 24). Sghiri et al. (22) reported ejaculation in only one out of 55 jacks (1.8%) despite using combinations of different imipramine (2 or 3 mg/kg) and xylazine (0.44 or 0.66 or 0.70 mg/kg) dosages, and different time intervals (1 or 2 h) between imipramine and xylazine administration. Mráčková et al. (19) reported ejaculation in zero out of 10 jacks (0%) using xylazine hydrochloride (0.66 mg/kg intravenously), and in two out of 10 jacks (20%) using detomidine hydrochloride (0.02 mg/kg intramuscularly) followed 15 min later by its half dose (0.01 mg/kg intramuscularly) in cases where the first dose did not result in ejaculation. In another study by the same group (24), a combination of imipramine (3 mg/kg orally) and xylazine (0.66 mg/kg intravenously), and a combination of butorphanol (0.02 mg/kg intravenously) and xylazine (0.33 mg/kg intravenously) were tested. However, ejaculation was reported in zero out of nine jacks (0%) with each of the two combinations. The differences in ejaculation rate between the studies might be attributed to a relatively higher dose of xylazine and younger age of the jacks (2–4 years) in the study by Naoman and Ali (23). Since the jacks in that study were housed under more controlled conditions and used in pilot trials before the actual study, it can also be presumed that the donkeys were more acclimatized and less stressed, which could have contributed to the higher ejaculation rate. Although PGF2α has not been tested for ex copula ejaculation in jacks, it has been shown to hasten the process of in copula semen collection by reducing the intervals to erection and ejaculation (25).

## Conclusions

Although pharmacologically induced ex copula ejaculation is a practical method for semen collection in stallions, a considerable variation in the ejaculatory rate has been reported between different protocols. There is also a significant individual variation between stallions. Some stallions respond to protocols involving xylazine while others respond to protocols involving detomidine. Therefore, multiple trials using different protocols should be performed in cases of failure to obtain semen in clinical situations. While the effects of prestimulation on ejaculation rate are inconsistent between protocols, a quiet environment with minimal disturbance during the pharmacological induction generally has a positive effect on the ejaculation rate. The comparable semen quality to in-copula ejaculations and the absence of serious side effects makes the pharmacologically induced approach a good alternative for stallions from which semen collection is not possible using the traditional approach. In contrast to the body of literature available on pharmacologically induced ex copula ejaculation in horses, there is very limited information on this topic in donkeys. Due to the limited information and inconsistent results observed in the few studies that have been conducted so far, it would be too early to determine if pharmacologically induced ex copula ejaculation is an effective method of semen collection in jacks. Further studies are warranted to evaluate and compare different pharmacological protocols for inducing ejaculation in jacks. Lastly, it might be worth exploring whether ex copula ejaculation can be used as an alternative method for semen collection in wild equids and other wild animal species.

## Author Contributions

AK and FK conceptualized the article. AK searched literature and wrote the first draft of the section on horses. AS searched literature and wrote the first draft of the section on donkeys. FK edited the drafts and prepared the final version of the manuscript. All authors contributed to the article and approved the submitted version.

## Conflict of Interest

The authors declare that the research was conducted in the absence of any commercial or financial relationships that could be construed as a potential conflict of interest.

## Publisher's Note

All claims expressed in this article are solely those of the authors and do not necessarily represent those of their affiliated organizations, or those of the publisher, the editors and the reviewers. Any product that may be evaluated in this article, or claim that may be made by its manufacturer, is not guaranteed or endorsed by the publisher.
